# Entry-Level Forward Surgical Team Training in 5th Grade Students of Second Military Medical University of the Chinese People’s Liberation Army

**DOI:** 10.1007/s00268-017-4035-2

**Published:** 2017-05-05

**Authors:** Shiguan Le, Wang Xi, Wei Li, Jian Xiao, Zhinong Wang

**Affiliations:** 1 Department of Cardiothoracic Surgery, Changzheng Hospital, Second Military Medical University, 31st Floor, 415#, Rd Fengyang, Shanghai, 200003 China; 2 Department of Cardiothoracic Surgery, 161 Hospital of Chinese People’s Liberation Army, Wuhan, 430012 China

## Abstract

**Background:**

Forward surgical team (FST) is a highly mobile team for surgical missions in battlefield. FST training has been well held in many western countries. However, such training in Chinese army is far from satisfaction.

**Methods and Results:**

Relying on Second Military Medical University and its affiliated hospitals, we are launching an entry-level training program for 5th grade students, in order to improve their understandings on basic concepts of FST, as well as their abilities to complete surgical missions on battlefield.

**Conclusions:**

In this article, we are going to introduce our training facilities as well as our training methods in our training program.

## Introduction

Forward surgical team (FST) is a 20- to 30-person medical unit that is highly mobile, which can perform surgical missions with relatively little need of outside support. It is comprised of general and orthopedic surgeons, nurse anesthetists, registered nurses, licensed practical nurses, operating room (OR) technicians, emergency medical technicians, and a healthcare administrator [1–3]. First established as Mobile Army Surgical Hospital (MASH) in Korean War, the unit was thought to be too cumbersome for effectively support in First Gulf War [4]. Therefore, an elite unit so-called FST was created and was developing in the following years and is now designed and equipped to perform surgery on as many as 30 wounded in a 72-h period before requiring resupply not only at the frontline of battlefield, but also of terrorist activities and natural disasters [1].

There are three main trauma training centers in America to train FSTs. The first was established at 2001 by the US Army, and then the US Navy and Air Force began their training programs [2]. All the three centers are established based on medical schools and focus on didactics, state-of-the-art simulation, and expeditionary equipment training specific to their mission, as well as actual clinical experience in the acute management of trauma patients. Usually, the FST training course is devised a 2-week program composed of a multimodality combination of lectures and laboratory exercises (Phase 1) and clinical experiences (Phases 2 and 3). And then, the trainee’s competency in FST function will be measured by several methods [5–7].

The current condition of combat casualty care in China is far from satisfaction. Though military medical schools have such courses as combat casualty care and military sanitation service, the training programs are not fit to real combat. Under such circumstances, we are trying to establish a new 30-day training program in military medical university for the 5th grade students, equals to the last year of the undergraduate program in Chinese medical education model, who have already finished and passed the theoretical courses of medicine and are with some basic surgical skills. Since these students would be deployed to different places of military medical services and chances are high that they will be selected for FST team in the future, the entry-level instructional program in this study is aimed at helping them understand basic concepts on FST, figure out the operating model and workflow, and master the fundamental treatment skills of war trauma.

In this training program, we will divide the students into different groups, and each group contains about 20 students. The role of each student will rotate in training process, in order to make all the students learn techniques of all the FST members. New training methods such as problem-based learning (PBL) and surgical simulators, as well as new evaluating tools, are going to be taken into this program to cultivate qualified FSTs.

In this article, we will introduce three main steps of FST training program in 5th grade students in Second Military Medical University and evaluate the effects of different methods, thus trying to optimize the best training method in China.

## Materials and methods

Before deployment, each 5th grade student participates in a 30-day training program at Center for Clinical Skills Training (CCST) of our university. The training program is divided into 3 phases. The primary aim of the program is to enhance the techniques of combat casualty care and to promote teamwork among team members. The training center comprises 15 staff, including 1 chief, 5 trainers, 5 support staff, 2 engineers and 2 veterinarians. All the staff are coming from the affiliated hospitals of our university. With these staff, five 20-person teams can be trained synchronously. Besides the 5th grade students, doctors in affiliated hospitals also participate, if space permits.

## CCST of Second Military Medical University

**Fig. 1:**
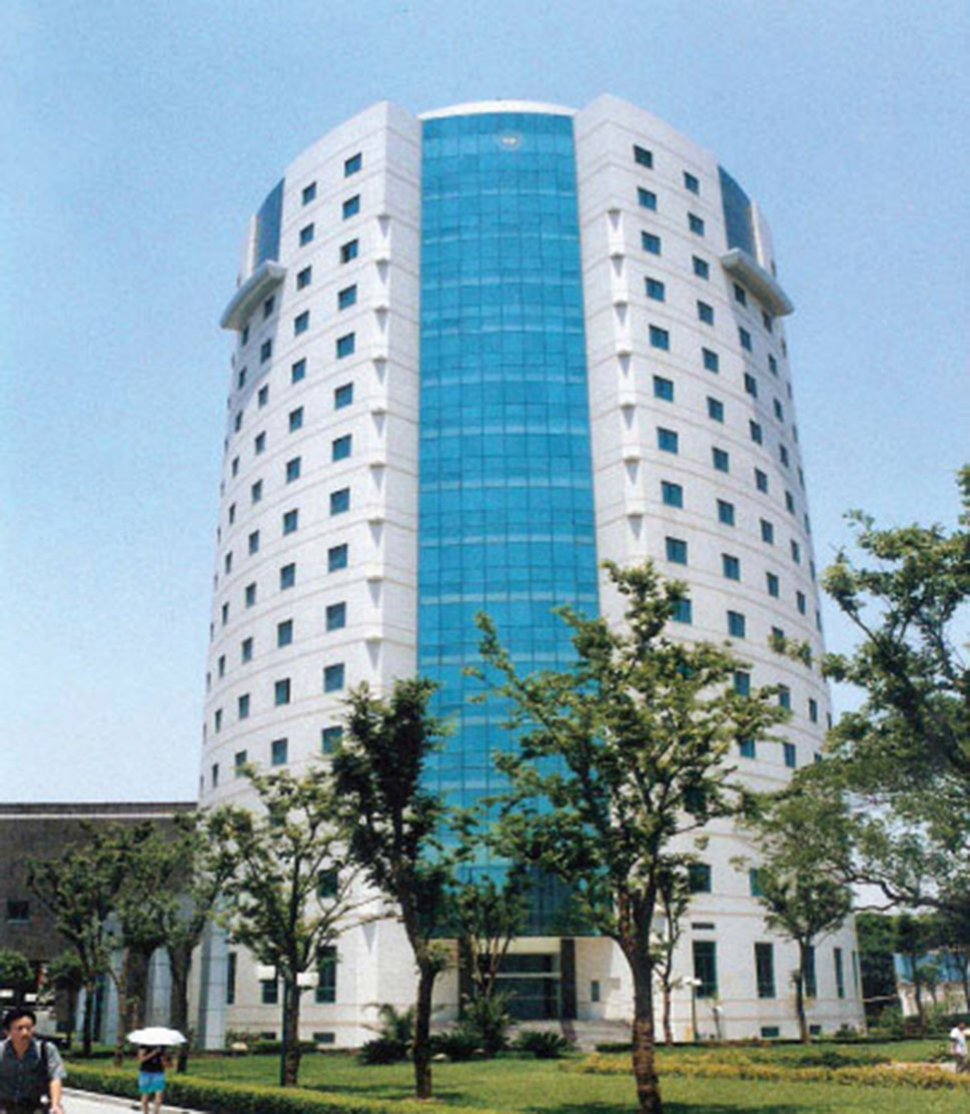
CCST of Second Military Medical School

**Fig. 2:**
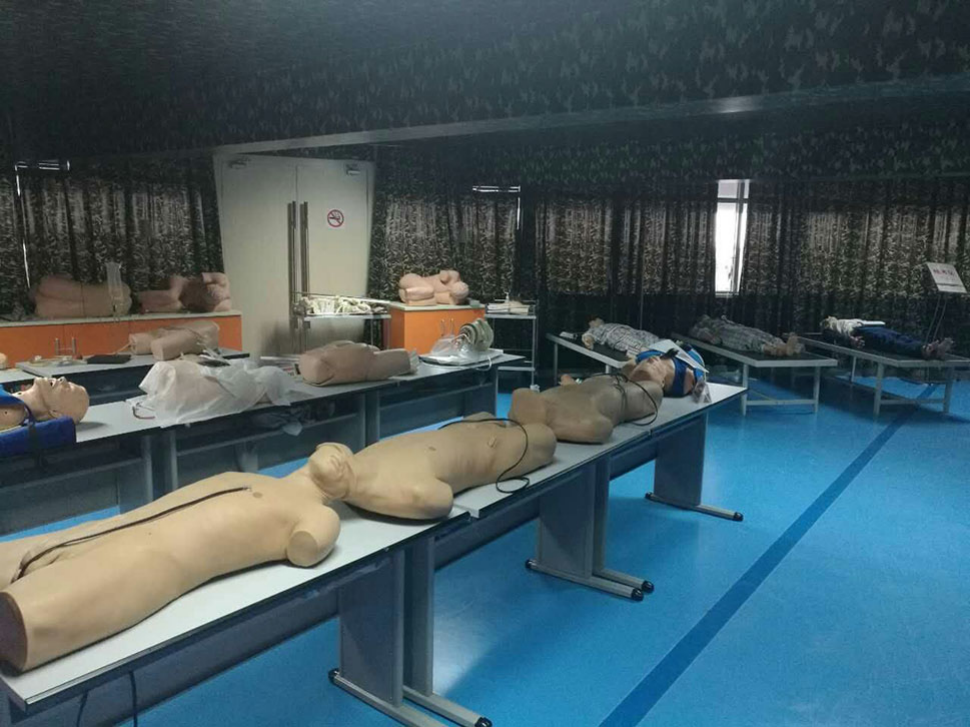
Mini center of CCST

**Fig. 3:**
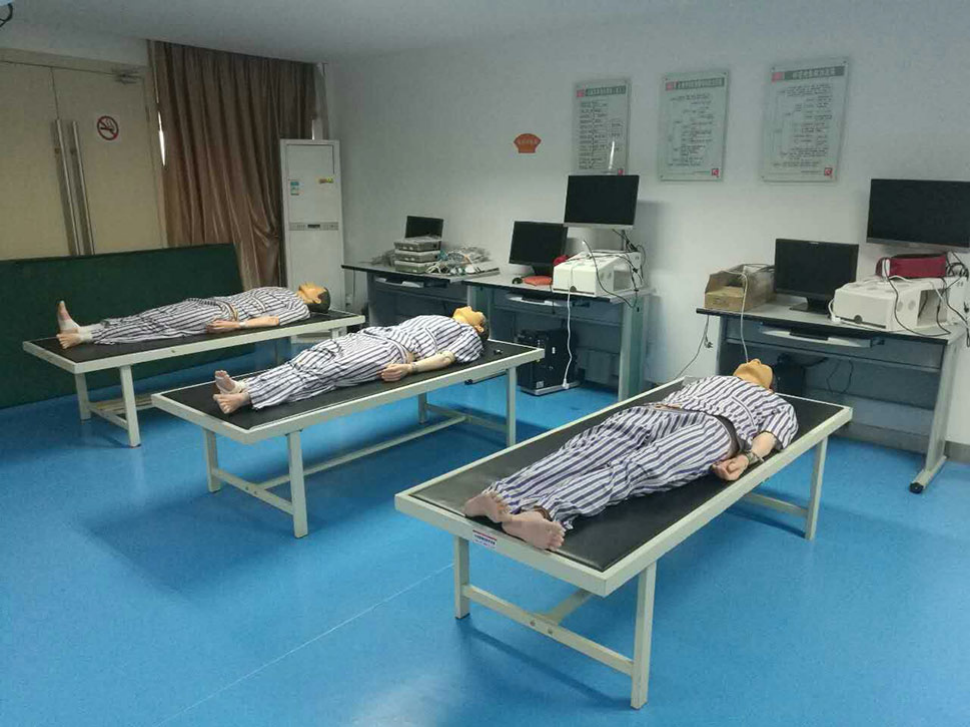
Mini center in CCST

The CCST of Second Military Medical University is the largest and the most sophisticated training center in China (Fig. 1). It is composed of several mini centers such as surgical skills training center, combat casualty care training center, FST environment training center and quality assessment center. Based on high-tech simulation equipment, all the training centers can build virtual battle terrains, so that the trainees can have immersive experience while training (Figs. 2, 3).

We have already created 15 combat casualty animal models according to the researches. We have also built libraries of Standard Wounded based on dogs, simulators and made the corresponding criteria, which will be discussed later.

## Phase one: didactic

The first 7 days of the program is theoretical knowledge study. In this phase, trainees will know the process and the rules of the program and the evaluation criteria. Trainees will also be taught the following subjects: some tactical combat casualty care (TCCC) theories, military sanitation theories, surgical skills and the use of FST equipment. Trainees will take nine 40-min lessons (4 in morning, 3 in afternoon and 2 after dinner) every day in this phase.

### Introduction of the program

At the first day trainees arrive the center, the staff of the center will guide them around the environment and to their rooms (usually 4 persons per room), where trainees live in the next 30 days.

The whole environment including special training equipment will be shown to trainees in the afternoon at the first day, and a program introduction will be made in the hall after visit. Then, all trainees will be divided into five 20-person groups, which will be trained as teams to perform surgical missions efficiently in battlefield.

Each group has a trainer and a support, who will introduce the courses to group members at day 2. Different characters will be distributed to each person of the group, and characters will be rotated regularly in the process.

### Study of TCCC and sanitation theories

TCCC theory is to guide and regulate the first-line medic-based casualty care and evacuation in battlefield. Different armies have different TCCC guidelines. We made our own teaching materials based on the handbook of TCCC of US Army and the former textbooks in Chinese Army.

Trainees will be taught how to observe the conditions and make right decisions in battlefield so that they can perform surgical missions as well as self and mutual medical aid optimally. They will learn how FSTs work, principles of triage, trauma team system, preoperative resuscitation unit, damage control surgery, perioperative care, severe multiple trauma control strategy and advanced trauma life support.

We will give some printed standard operation procedures (SOPs) on massive hemorrhage, heart arrest, asphyxia, pneumothorax, shock, hypothermia, burn, fracture and pain to the trainees, so that they can carry out missions following such procedures. All procedures were made based on the update guidelines by experts in our university.

### Study of advanced surgical skills

**Fig. 4:**
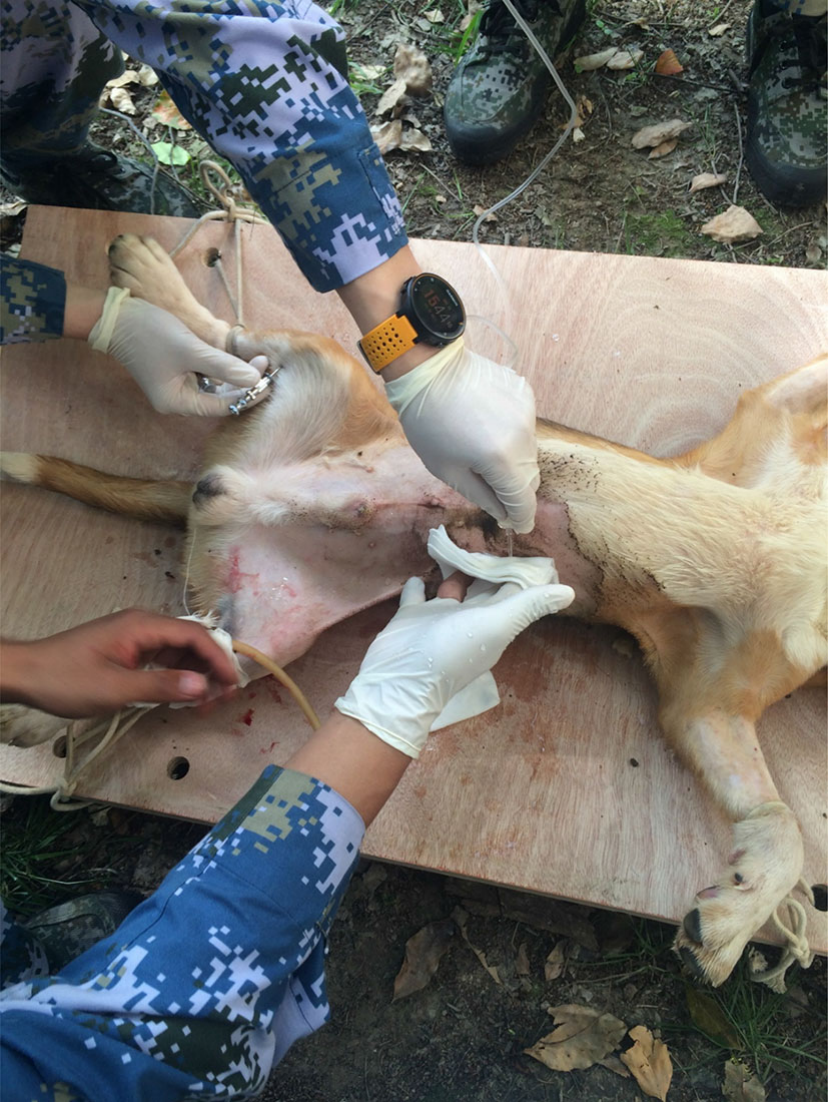
Making injury models on dogs

The US Army have listed 57 surgical procedures among 350 injuries, which are classified by different regions such as head and neck, upper and lower extremity, chest, abdomen/pelvis, and burns [8–10]. In Chinese Army, 22 surgeries are listed in FST operation procedure and divided into two levels: emergency life-saving surgery and damage control surgery (Fig. 4).

Trainees will be able to perform amputation, vessel repair, tracheotomy, open pneumothorax closure, closed drainage of pneumothorax, exploratory thoracotomy, peri-cardiocentesis, extracorporeal membrane oxygenation, exploratory laparotomy, craniotomy, extremity repair, packing hemostasis in parenchyma organ injuries, hemostasis and bandaging in extremity, ligation, compartment syndrome decompression, severe open fracture fixation, debridement, hollow organs clamping, colostomy, lienal rupture repair, liver rupture repair and firearm injury debridement in proper time after training in our center (Table 1).

**Table 1:** Surgical types and time probability distribution of forward surgical team

Category	Surgical types	Median operation time (min)
Life-saving surgery	Amputation	45
	Vessel repair	60
	Tracheotomy	20
	Open pneumothorax closure	15
	Closed drainage of pneumothorax	9
	Exploratory thoracotomy	95
	Peri-cardiocentesis	35
	Extracorporeal membrane oxygenation	25
	Exploratory laparotomy	60
	Craniotomy	130
	Extremity repair	30
Damage control surgery	Packing hemostasis in parenchyma organ injuries	45
	Hemostasis and bandaging in extremity	10
	Ligation	20
	Compartment syndrome decompression	20
	Severe open fracture fixation	45
	Debridement	60
	Hollow organs clamping	20
	Colostomy	20
	Lienal rupture repair	45
	Liver rupture repair	60
	Firearm injury debridement	60

They will also have the capacity for anesthesia, ICU nursing care, triage and evacuation, as well as special situation awareness and coordination in different tactical environment.

In this section, different surgical skills will be demonstrated by multimedia and performed by trainers on simulators to trainees, and SOPs will be explained to them as well. Practice will be arranged in Phase 2.

A theoretical examination will be held at the end of this phase. Anyone who did not passed the examination will not move on to Phase 2. They will have another chance to pass the exam at the next day, or they are going to relearn the theoretical knowledge.

## Phase two: practice

In this phase, it will take 20 days for trainees to learn and practice all the techniques of FSTs. Each person of a training group will be divided into three small sections: advanced life support section, operation room section and recovery section. Sections will rotate every 6 days. Trainer and support are responsible for each group’s training, administration and evaluation. In our training center, PBL is used throughout this phase. Different conditions will be set for trainees, and trainers will guide them to learn techniques while solving those problems.

### Special FST equipment

The FST is highly mobile and operates in a tent shelter which has about 300–400 m^2^ of functional space. An FST in China owns one surgical operating shelter vehicle and six modified Chinese Humvee, and all facilities are highly consolidated and packaged on these vehicles, with which FST can start surgery in 90 min and evacuate in 60 min. All facilities are modularized designed into three sections, which are call life supplement section, damage control treatment section and perioperative care section. Each section is divided into different groups. Details of these sections and groups are listed in Table 2.

**Table 2:** FST equipment

Section	Group	Equipment
Life supplement	Basic installation	Tent *3
	Basic equipment	Sickbed *4, operating table *2, stretcher *6, oxygen cylinder *5
Damage control	Life support	Portable ventilator *2, Vscan *2, life support case *2 (Tracheotomy suite, intubation suite, Intravenous transfusion suite, intraosseous transfusion suite, CRBC, medicines), Triage suite *2
	Surgery	Portable anesthesia machine *2, portable monitor *3, general surgery case *2, orthopaedics surgery case *2, anesthesia case *2, surgical consumables case *5
Perioperative care	ICU	Portable ventilator *4, portable monitor *4, portable blood analyzer *1, ICU specific medical case *1

**Fig. 5:**
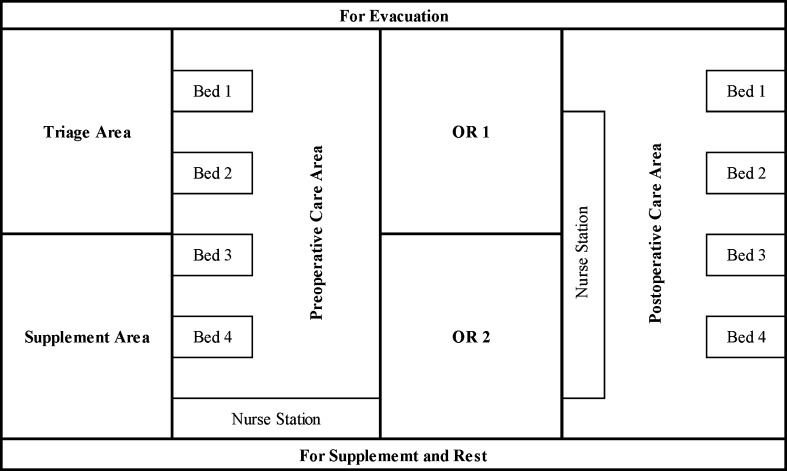
Possible layout of a forward surgical team

In combat, we divided the shelter into 6 independent but interconnected places for triage, operation, ICU, recovery, rest and supplement area, which is shown in Fig. 5.

### Scenario simulation

As our training is based on different traumas, trainees have opportunities for training in volunteer-mock patients, experimental dogs and advanced simulators.

A component of the training is to use costumed volunteers, who have been briefed on their clinical manifestations based on their simulated condition. We have built 150 different simulated conditions. We also introduce Human Worn Partial Task Surgical Simulator (Cut-Suit) for better effect. The team is alerted to one or more incoming wounded, and the team must carry out an optimal triage at once and then treat the wounded following protocols and guidelines. The drill is performed under the observation of trainers, and the treating process will be recorded and marked. Trainers can cease the treating process at any time when problems occur and guide the trainees to solve them properly.

Simulated volunteers are always used to enhance the capacities for triage, first aid, and ICU nursing care, while experimental dogs are used to improve the surgical skills.

We have created 15 combat casualty Beagle models based on different regions or causes of injuries, which will be performed surgeries on. These models include bleeding in extremity, fracture, blast injuries and so on. All the procedures are approved by the committee on ethics of biomedicine research of our university and followed by the “Regulations for the Administration of Affairs Concerning Experimental Animals”.

Usually, a surgical team comprises five trainees, including one anesthetist, two surgeons, one scrub nurse and one circulating nurse, and the characters will rotate per surgery. Before surgery, our staff will build different models following protocols, which will be distributed to each surgical team. Since the trainees are 5th grade students who have finished their internship in affiliated hospitals, and they have been demonstrated all the surgical skills in Phase 1, we will first leave these models to them to record their reactions and to test their abilities. Our trainers will tell them details or techniques they are going to use in the surgery and guide them throughout the whole process of the surgery (Fig. 5). Taken charge by trainers, the surgical team will discuss the pros and cons in the procedure after surgery, whether it is a successful or unsuccessful treatment. It is such a seminar or brainstorming that either techniques, or surgical skills or teamwork can be discussed so that not only the abilities of trainees will improve, but the surgical protocols can also be optimized. Reports of each group for submission to the chief of the training center will be discussed in the staff meeting every night, in order to improve the pedagogics and adjust the process of training. Trainees will perform two or three surgeries in 1 day, and study lounges are arranged after dinner for trainees to review the skills and knowledge they have learned in daytime.

After trainees have good command on every surgical skills and other techniques, they will have to face and solve mass casualty incidents in complicated environment, which is created by our simulators. They will act as real FSTs in our simulating laboratories to save the wounded built by simulators.

In our training center, we have introduced several sophisticated simulators, such as Human Patient Simulator (HPS-7909, CAE Healthcare), Emergency Care Simulator (ECS-7910, CAE Healthcare), SimMan^®^ 3G Trauma (Laerdal Medical), ship motion simulator for animals (R&D in our university), human 6° motion ship simulator (R&D in our university), battlefield environment simulator and virtual reality training system. They are integrated with computer software that let them replicate abnormal bodily responses to different injuries and therapeutic interventions. Trainees can perform not only trachea intubation, gastric tube incision, CPR and venous catheterization, but they can also carry out some advanced treatment such as tracheotomy, closed drainage of pneumothorax, peri-cardiocentesis and so on.

**Fig. 6:**
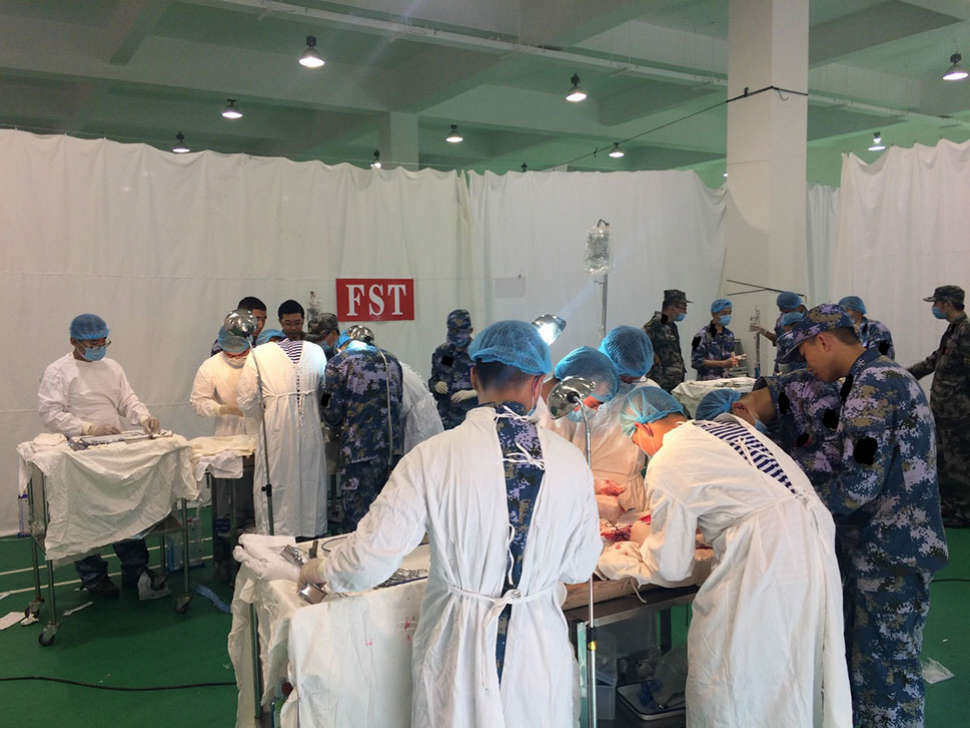
Performing surgeries in simulating scenarios

In addition to those training programs, we have created some new scenes based on real battlefield, comprised of immersive noise, light, smoke, gun shot and different enemy status. It is a dynamic environment for members of a surgical team to cooperate with each other to save the wounded. Trainee will also have chance to perform surgery in moving vehicles or ships, and other tough circumstances. They are going to experience the real scene, so that they can improve their competence of teamwork skills, leadership, situation awareness, decision making, coordination as well as technique skills (Fig. 6).

In this section, a team leader is chosen to command the whole mission. He/she is going to lead the FST, a 20-person medical team, to make the right assignment, to do the right decision and to complete the mission. The leader will rotate in each mission.

Trainers will observe the whole training process and mark the mistakes that trainees have made. After training, usually two subjects a day, a debriefing will be organized by the trainers, and the leaders of the two missions are going to give their reports on their teams, which are not circumscribed. After that, the trainers will point the mistakes that trainees made in the mission and guide the trainees to solve the mistakes themselves by discussion. The trainers will make a conclusion to the mission at the end of the debriefing.

## Phase three: evaluation

In this phase, a 3-day rehearsal will be held to test the trainees’ abilities. Trainers and experts are going to observe the performance of all the trainees, and do in-depth quantitatively assessment through different methods. We have designed several standardized situations for trainees to solve, in order to score their performance.

### Objective Structured Assessment of Technical Skills (OSATS)

The Objective Structured Assessment of Technical Skills (OSATS) is a famous examination since 1990s, which is comprised of an operation-specific checklist and a global rating scale [11]. It has already been proved feasible and effective to assess the surgical skills of trainees [12]. In our training center, each trainee will be scored the surgical skills using the global rating scale of OSATS, which consisted of 35 points on 7 items, to assess the qualification for deployment (Table 3).

**Table 3:** Global rating scale of operative performance. Please circle the number corresponding to the trainee’s performance in each category, irrespective of training level

	1	2	3	4	5
Respect for tissue	Frequently used unnecessary force on tissue or caused damage by inappropriate use of instruments		Careful handing of tissue but occasionally caused inadvertent damage		Consistently handled tissue appropriately with minimal damage
	1	2	3	4	5
Time and motion	Many unnecessary moves		Efficient time/motion but some unnecessary moves		Clear economy of movement and maximum efficiency
	1	2	3	4	5
Instrument handling	Repeatedly makes tentative or awkward moves with instruments by inappropriate use of instrument		Competent use of instruments but occasionally appeared stiff or awkward		Fluid moves with instruments and no awkwardness
	1	2	3	4	5
Knowledge of instruments	Frequently asked for wrong instrument or used inappropriate instrument		Knew names of most instruments and used appropriate instrument		Obviously familiar with the instruments and their names
	1	2	3	4	5
Flow of operation	Frequently stopped operating and seemed unsure of next move		Demonstrated some forward planning with reasonable progression of procedure		Obviously planned course of operation with effortless flow from one move to the next
	1	2	3	4	5
Use of assistants	Consistently placed assistants poorly or failed to use assistants		Appropriate use of assistants most of time		Strategically used assistants to the best advantage at all time
	1	2	3	4	5
Knowledge of specific procedure	Deficient knowledge. Needed specific instruction at most steps		Knew all important steps of operation		Demonstrated familiarity with all aspects of operation

### Imperial College Assessment of Technical Skills for Nurses (ICATS-N)

Though the trainees are mostly majored in doctor of medicine, they need to be able to play different roles in FSTs as the lack of medical officer in battlefield still exists. One important role is being a nurse in the operation room, especial scrub nurse [13]. In our training program, all trainees will be trained how to be a qualified nurse. Referring to the methods how US Army trains the surgical team [5], we are going to use Imperial College Assessment of Technical Skill for Nurse (ICATS-N) to assess the skills of the trainees [14], which is gowning and gloving, setting up instrumentation, draping, and maintaining sterile field (Table 4).

**Table 4:** Imperial College Assessment of Technical Skills for Nurses (ICATS-N)

Core skill	Observable items	Scale						
Gowning and gloving	Al. Gowning and gloving using closed method	N/A	1	2	3	4	5	6
	A2. Placement of gloved hand: clasped mid-chest	N/A	1	2	3	4	5	6
	A3. Back of gown closed using tag	N/A	1	2	3	4	5	6
Setting up instrumentation	B1. Established working area	N/A	1	2	3	4	5	6
	B2. Individually count and name instrument with circulating nurse	N/A	1	2	3	4	5	6
	B3. Count swabs in 5s, showing Raytex and tie	N/A	1	2	3	4	5	6
	B4. Placement of sharps in kidney dish	N/A	1	2	3	4	5	6
	B5. Prepare swabs for cleaning	N/A	1	2	3	4	5	6
Draping	C1. Ensure 2 team members drape together	N/A	1	2	3	4	5	6
	C2. Hand drape over right-side up and without dragging (supporting drape); open tray drape in sterile manner	N/A	1	2	3	4	5	6
	C3. First two sides, followed by bottom, followed by top	N/A	1	2	3	4	5	6
Maintaining sterile field	D1. Hand instrumentation to surgeon in a sterile manner without touching working end	N/A	1	2	3	4	5	6
	D2. Anticipate surgeon’s needs (e.g., clip-clip-scissors-ties, suction, larger swabs)	N/A	1	2	3	4	5	6
	D3. Have control of instrumentation and soiled swabs—i.e., no instruments lying on top of patient	N/A	1	2	3	4	5	6
	D4. Posture and movement: when facing the trolley keep eye contact on procedure	N/A	1	2	3	4	5	6

N/A, Not applicable; 1, Not done at all; 6, Done very well

### Non-technical skills (Oxford NOTECHS)

An FST is such a team that can accomplish difficult rescue missions in battlefield. The success depends not only on magnificent technical skills but also an efficiency teamwork, which needs all team members have the ability to generate positive chemical reactions with each other in a very short period. We use a theater team non-technical skills scoring system call Oxford NOTECHS to assess trainees’ teamwork behavior [15, 16] (Table 5).

**Table 5:** Operating-theater team non-technical skills assessment tool

Leadership and management	
Leadership	Involves/reflects on suggestions/visible/accessible/inspires/motivates/coaches
Maintenance of standards	Subscribes to standards/monitors compliance to standards/intervenes if deviation/deviates with team approval/demonstrates desire to achieve high standards
Planning and preparation	Team participation in planning/plan is shared/understanding confirmed/projects/changes in consultation
Workload management	Distributes tasks/monitors/reviews/tasks are prioritised/allots adequate time/responds to stress
Authority and assertiveness	Advocates position/values team input/takes control/persistent/appropriate assertiveness
Teamwork and cooperation	
Team building/maintaining	Relaxed/supportive/open/inclusive/polite/friendly/use of humor/does not compete
Support of others	Helps others/offers assistance/gives feedback
Understanding team needs	Listens to others/recognizes ability of team/condition of others considered/gives personal feedback
Conflict solving	Keeps calm in conflicts/suggests conflict solutions/concentrates on what is right
Problem solving and decision making	
Definition and diagnosis	Uses all resources/analytical decision making/reviews factors with team
Option generation	Suggests alternative options/asks for options/reviews outcomes/confirms options
Risk assessment	Estimates risks/considers risk in terms of team capabilities/estimates patient outcome
Outcome review	Reviews outcomes/reviews new options/objective, constructive and timely reviews/makes time for review/seeks feedback from others/conducts post-treatment review
Situation awareness	
Notice	Considers all team elements/asks for or shares information/aware of available of resources/encourages vigilance/checks and reports changes in team/requests reports/updates
Understand	Knows capabilities/cross-checks above/shares mental models/speaks up when unsure/updates other team members/discusses team constraints
Think ahead	Identifies future problems/discusses contingencies/anticipates requirements

*Below standard* behavior directly compromises patient safety and effective teamwork, *Basic standard* behavior in other conditions could directly compromise patient safety and effective teamwork, *Standard* behavior maintains an effective level of patient safety and teamwork, and *Excellent* behavior enhances patient safety and teamwork; a model for all other teams

## Conclusions

Our training center was established at 2010. To date, about 1500 students, surgeons and nurses have been trained in our center, and most of them are now playing important roles in the mobile forces of medical service so-called FSTs in China. To date, we have followed some of our students. The feedback showed that the effect is positive and our students could incorporate into the team more smoothly. With the development of our training center, we designed lots of advanced training facilities, created standardized battlefield situations and set normalized injuries. But so far, our training program is designed for the entry-level undergraduates. Though the training effect is proved good in natural disaster rescue missions, the effect in battlefield remains unclear. But what remains certain is that such training in Chinese military medical university need to be continued, and further research on “consultant-level” or “master-level” continuity program need to be carried on in the future.
